# Effect of Graphene Oxide on the Low-Temperature Crack Resistance of Polyurethane–SBS-Modified Asphalt and Asphalt Mixtures

**DOI:** 10.3390/polym14030453

**Published:** 2022-01-23

**Authors:** Shuai Li, Wenyuan Xu, Fengfa Zhang, He Wu, Pengchao Zhao

**Affiliations:** 1 College of Civil Engineering, Northeast Forestry University, Harbin 150040, China; ls15729068066@163.com (S.L.); dapeng_zpc@sina.com (P.Z.); 2 Heilongjiang Institute of Technology, College of Civil Engineering, Harbin 150050, China; ls18845129096@163.com (F.Z.); hgcwh@163.com (H.W.)

**Keywords:** graphene oxide modifier, roadworks, modified asphalt, low-temperature resistance, OpenCV graphics technology

## Abstract

In this study, the novel nanomaterial graphene oxide (GO) was added as a modifier to polyurethane–styrene-butadiene-styrene (SBS)-modified asphalt, and a graphene oxide/polyurethane/SBS composite-modified asphalt mix was prepared. The effect of the graphene oxide material on the low-temperature crack resistance of the asphalt and mixes was investigated by bending beam rheometer (BBR) tests, beamlet bending tests at different low temperatures, and characterization by scanning electron microscopy for its microscopic condition. OpenCV image processing was used to visually represent the low-temperature cracking of the mix. The results of the BBR tests showed that the incorporation of graphene oxide resulted in a reduction in creep stiffness *S* and an increase in creep rate *m* compared with the control asphalt. The best improvement in the low-temperature cracking resistance of the polyurethane/SBS-modified asphalt was achieved at 0.5% GO doping. The results of the small beam flexural tests showed that graphene oxide as a modifier improved the flexural strength and flexural strain of the mix, resulting in a mix with a lower stiffness modulus and a better relaxation stress capacity with the addition of graphene oxide, which is also expressed through the OpenCV images. Graphene oxide significantly improved the low-temperature crack resistance of polyurethane-SBS-modified asphalt and its mixes. As a new type of nanomaterial-modified asphalt, graphene oxide/polyurethane/SBS composite-modified asphalt shows promising applicability in cold zone roads.

## 1. Introduction

With the prolongation of road service, asphalt pavement distress has gradually deteriorated in recent years due to problems such as rutting, cracking, and spalling [1,2,3]. Among other things, climate and environmental changes, especially low temperatures, have a serious impact on asphalt pavements, reducing the life cycle of asphalt pavements, increasing road maintenance costs, and significantly impacting the high quality level of road service [3,4]. Asphalt modification is considered to be a practical solution to the low-temperature fracture of asphalt materials, improving the low-temperature performance of asphalt pavements [5]. With the development of nanotechnology, nano-modified asphalt has become a new research hotspot in the field of road transport materials. Graphene oxide (GO) is a single atomic layer formed by the oxidation of graphite, with a unique quasi-dimensional layered structure, excellent oxygen barrier, and good intersolubility with organic solvents, gaining attention as a new modifier for asphalt [6,7,8]. Research in recent years has found that graphene oxide as a modifier can improve the elastic response and road performance of the asphalt matrix, forming a more stable molecular structure [9,10]. In addition, the cost of nanomaterials has shown a decreasing trend, and with improvements in manufacturing technology, their costs are likely to decrease further [11]. This would improve the scalability of graphene oxide as an asphalt modifier to improve asphalt road performance.

Zhang and other researchers tested the performance of styrene-butadiene-styrene-modified asphalt using both domestic and Superpave test methods and found that the addition of SBS modifiers improved the high-temperature performance but reduced the temperature sensitivity of the asphalt binder [12]. Yu et al. used thermoplastic polyurethane (TPU) as a reactive polymer modifier and found that the TPU functional group played a role in improving the thermal properties, high-temperature storage stability, and dispersion of the modified asphalt, but improvements in the low-temperature properties of the asphalt were not very significant [13]. In their study of the properties of the polyurethane/graphene oxide (PU/GO) nanocomposites prepared from graphene oxide, Yu et al. found that GO polymer particles showed good dispersion in the asphalt system. A synergistic effect of the polymer and nano-modification was achieved in the dispersion of modified asphalt materials to improve the performance of the materials [14]. Chen et al. prepared modified bitumen using waste polyurethane (WP) instead of styrene-butadiene-styrene (SBS) modifiers. They found that the addition of polyurethane improved the high- and low-temperature performance of SBS-modified asphalt [3].

Although many studies have shown that the addition of polyurethane or graphene oxide can improve the low-temperature performance of asphalt, most studies have not used a systematic research approach to demonstrate that the incorporation of these modifiers can form a steady-state structure that improves the low-temperature cracking performance of asphalt and mixes [15,16,17,18,19]. Meanwhile, the aforementioned researchers found that the addition of SBS and TPU did not improve the low-temperature performance and temperature-sensitive properties of the composite-modified asphalt very well. In this study, graphene oxide (GO) was added as a modifier to thermoplastic polyurethane (TPU)–SBS-modified bitumen, and a modified bitumen mix was prepared. Bending rheometer (BBR) tests and low-temperature bending tests on small beams at different temperatures were used from the asphalt and mix and the micro-level perspectives. The obtained results were then image-processed using OpenCV image processing technology to demonstrate the improvement of GO on the low-temperature crack resistance of TPU–SBS asphalt in a more intuitive and systematic way. This study provides more options for the use of asphalt pavements for roads in cold regions.

## 2. Materials and Methods

### 2.1. Raw Materials and Preparation of Samples

#### 2.1.1. Asphalt Raw Materials

**Figure 1:**
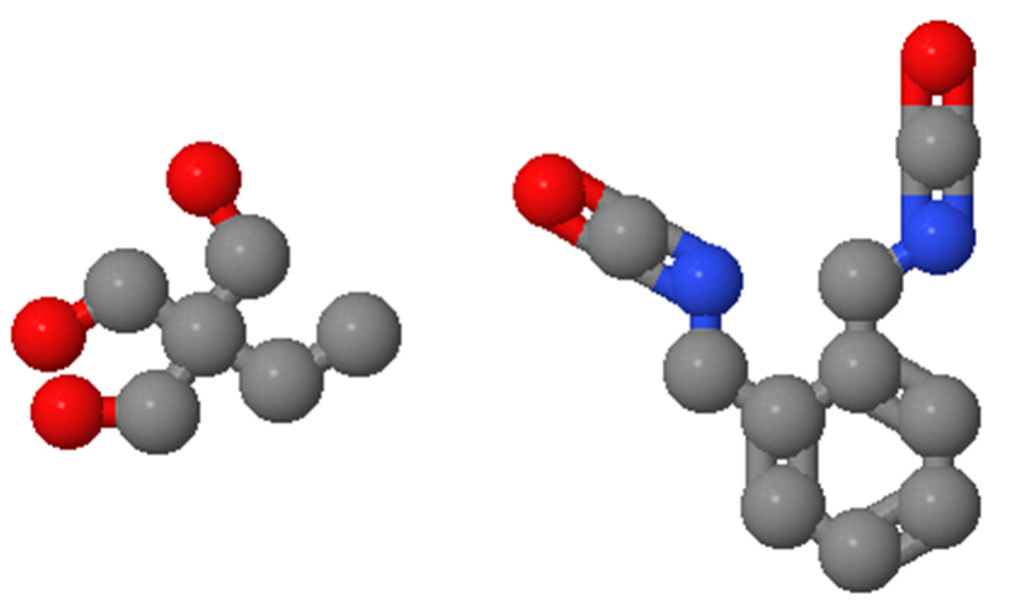
3D structure of TPU particles.

**Figure 2:**
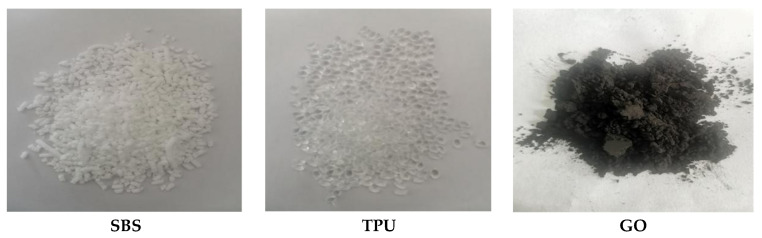
Appearance of the modifiers used in this research.

No. 90 matrix asphalt, originating from Panjin, China, was used as the matrix asphalt, and its basic technical specifications are shown in Table 1 (technical specifications were tested according to the Standard Test Method for Asphalt and Asphalt Mixture for Highway Engineering (JTG E20-2011)). The SBS used was SBSYH-792E thermoplastic styrene-butadiene rubber, produced by Sinopec, with a star structure, the basic indicators of which are shown in Table 2. Thermoplastic polyurethane granules (TPU) of German origin were used, and their 3D structural formula is shown in Figure 1. The basic specifications of graphene oxide (GO) are shown in Table 3. (The external appearance of the modifier is shown in Figure 2).

#### 2.1.2. Preparation of TPU–SBS-Modified Asphalt

TPU–SBS-modified asphalt was prepared by the melt blending method, mainly using asphalt mixers and high-speed shears to disperse the polyurethane particles and SBS modifiers in the asphalt. First of all, the 90-base asphalt was placed in a metal vessel and heated in a constant-temperature oven at 150 °C for 2 h until it became fluid. Then, the molten asphalt was placed on an electric heating plate at 160 °C, and the SBSYH-792E modifier and TPU modifier were added in turn. The asphalt mixer was switched on and stirred at 300 r/min for 30 min until there were no obvious solid particles. Next, the high-speed shear with a rotor speed set at 3000 r/min was used to shear at 160 °C for 45 min at a high speed to obtain TPU–SBS-modified asphalt after the full reaction.

The SBSYH-792E modifier was blended at 4.5% and the TPU modifier was blended at 5%. Based on previous experimental studies, the road performance of the asphalt was found to be proportionally optimal when the SBSYH-792E and TPU modifiers were dosed at 4.5% and 5%, respectively.

#### 2.1.3. Preparation of GO–TPU–SBS-Modified Asphalt

GO–TPU–SBS-modified asphalt was prepared by adding the nanomaterial modifier GO to the prepared TPU–SBS-modified asphalt using a high-speed shear.

#### 2.1.4. Raw Materials for Asphalt Mixes

Aggregates were selected from limestone specification aggregates and machine-made sand produced at Yuchuan Quarry in Acheng District, Harbin, China. The grade of the AC-16 asphalt mix used for the test is shown in Table 4. (The technical specifications for aggregate classification are given with reference to GTGF40-2004.). Asphalt was used as previously described. The bitumen content of the mixture was 4.3%.

### 2.2. Test Methods

Scanning electron microscopy (SEM) and bending rheometer tests (BBR) were carried out on GO–TPU–SBS-modified asphalt and TPU–SBS-modified asphalt. AC-16 was used as the mix structure type, and low-temperature trabecular bending tests were carried out for different temperature scenarios. The low-temperature crack resistance of asphalt and asphalt mixes were tested, and the test results were designed to be processed using OpenCV image technology to provide a better visual representation of the test results. (The test methods refer to the Standard Test Methods for Asphalt and Asphalt Mixtures for Highway Engineering (JTG E20-2011)).

#### 2.2.1. SEM Tests

Bench-top scanning electron microscopy (SEM, EM30/EM-30plus, COXEM, Korea) is an important research method used in materials science to observe the microscopic morphology and structure of materials [20]. To observe the effect of GO on the microscopic characteristics of TPU–SBS-modified asphalt, the scanning electron microscope was operated at 5 kV with a magnification of 100–600 times. Before the specimens were observed, they were continuously sputter-coated with a thin gold film and placed under infrared light for drying [21].

#### 2.2.2. BBR Test

**Figure 3:**
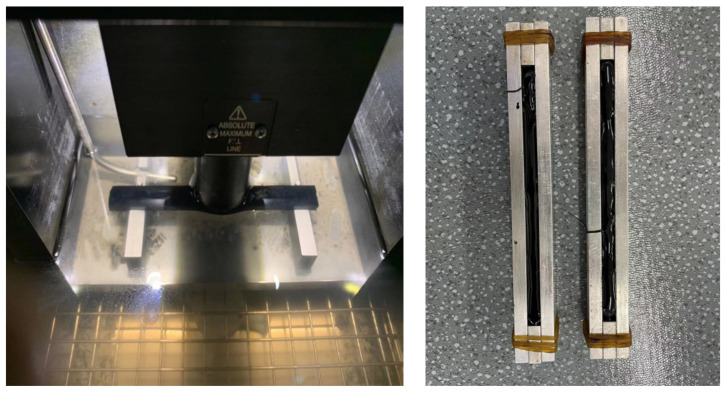
Diagram of the BBR test process.

BBR (TE-BBR-F, CANNON, USA) tests were used to evaluate the effect of GO on the low-temperature crack resistance of TPU–SBS-modified asphalt at −16 °C and −20 °C. (The BBR test apparatus is shown in Figure 3.) The creep stiffness *S* and creep rate *m* were calculated from the load and deformation values obtained over 60 s. The low-temperature cracking resistance of the modified asphalt was evaluated based on the calculated creep stiffness *S* and creep rate *m* [22,23]. Based on the data obtained from the tests, the GO dosage level for the best improvement of the low-temperature crack resistance of the TPU–SBS-modified asphalt was achieved.

#### 2.2.3. Low-Temperature Bending Test for Small Beams

**Figure 4:**
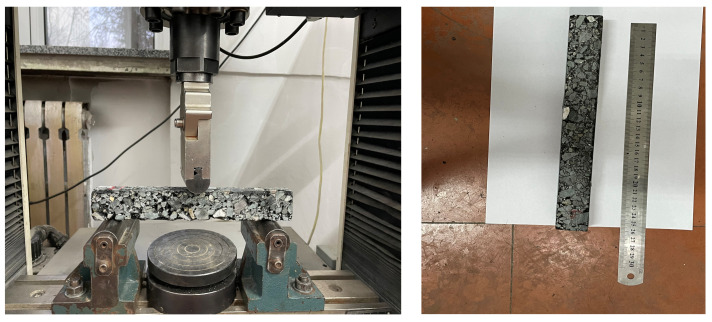
Process diagram of the low-temperature bending test for small beams.

The low-temperature bending test is a simple and effective method used to study the low-temperature fracture resistance of asphalt mixtures.(The low temperature bending test apparatus is shown in Figure 4.) To investigate the improvement of GO on the low-temperature fracture performance of asphalt mixes, the test temperatures of 0, −5, −15, and −25 °C were chosen for the asphalt mixes, considering the working temperature of asphalt pavements in the winter in cold regions. According to the JTG E20-2011 standard, low-temperature beam bending tests were carried out on two asphalt mixes with and without a GO dosing of 0.5% [24].

First, the rutting slabs were cut into 250 mm × 30 mm × 35 mmtrabecular specimens using a rock cutter, according to the specifications. Then, the small beam specimens were individually placed in a modulated temperature incubator for 3 h. Finally, the beams were tested by bending at room temperature with a set loading rate of 50 mm/min.

#### 2.2.4. OpenCV Image Processing

Computer vision is one of the most popular application areas of artificial intelligence [25]. The introduction of computer vision for image processing in material science has become a promising trend. In this study, the low-temperature cracking process of asphalt mixtures doped and un-doped with GO was processed by OpenCV pixel subtraction using the Python programming language for small beam bending test processes to more clearly and graphically represent the process.

## 3. Results and Discussion

### 3.1. Apparent Morphology

**Figure 5:**
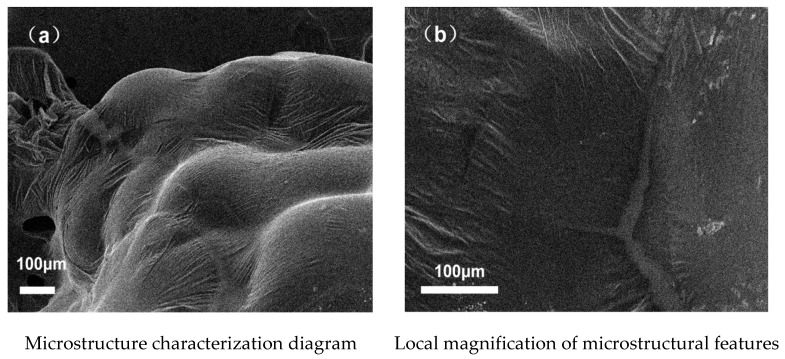
Apparent characteristics of asphalt without GO.

**Figure 6:**
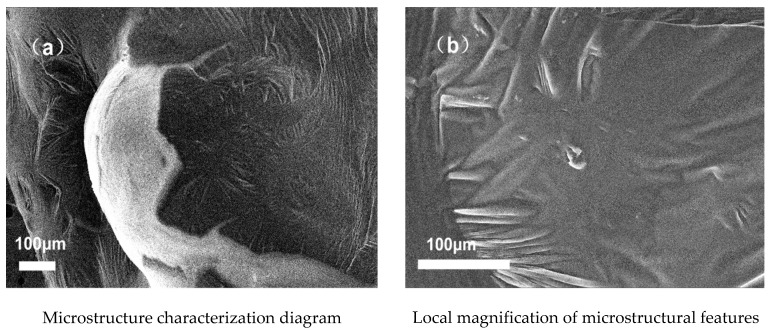
Apparent characteristics of asphalt mixed with GO.

SEM analysis of the structural characteristics of TPU–SBS-modified asphalt with and without the addition of GO provided the experimental results shown in Figure 5 and Figure 6. Figure 5a shows the microstructural characteristics of TPU–SBS-modified asphalt without the addition of GO, and Figure 5b shows the local enlarged form of Figure 5a. Figure 6a shows the microstructure of the TPU–SBS-modified asphalt doped with GO, and Figure 6b shows a partial enlargement of Figure 6a. From the results, it can be seen that the doping of the nanomaterial modifier GO can form a needle-like lamellar structure in the asphalt. Additionally, comparing plots in Figure 5 and Figure 6, it is evident that the doping of GO increased the degree of surface folding of the asphalt matrix, i.e., graphene oxide is present in the composite as folded flakes [26].

### 3.2. Effect of Graphene Oxide (GO) on the Low-Temperature Performance of TPU–SBS Asphalt

**Figure 7:**
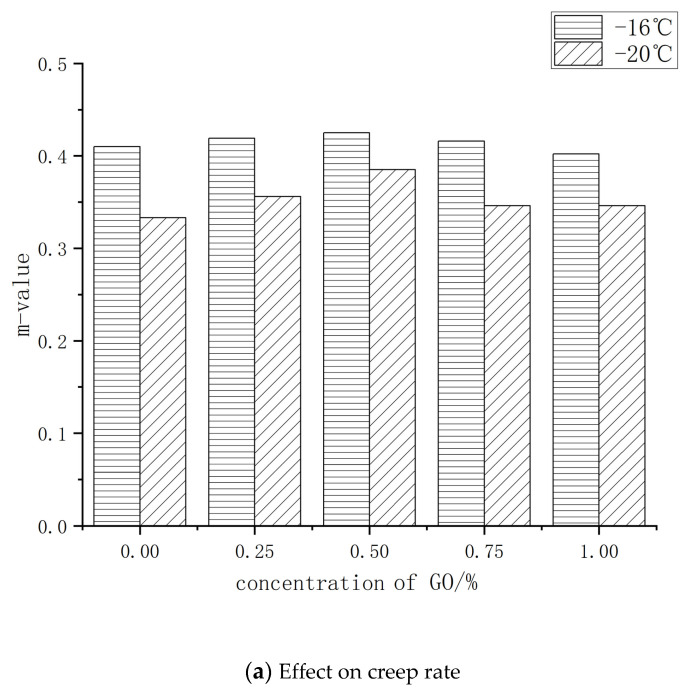

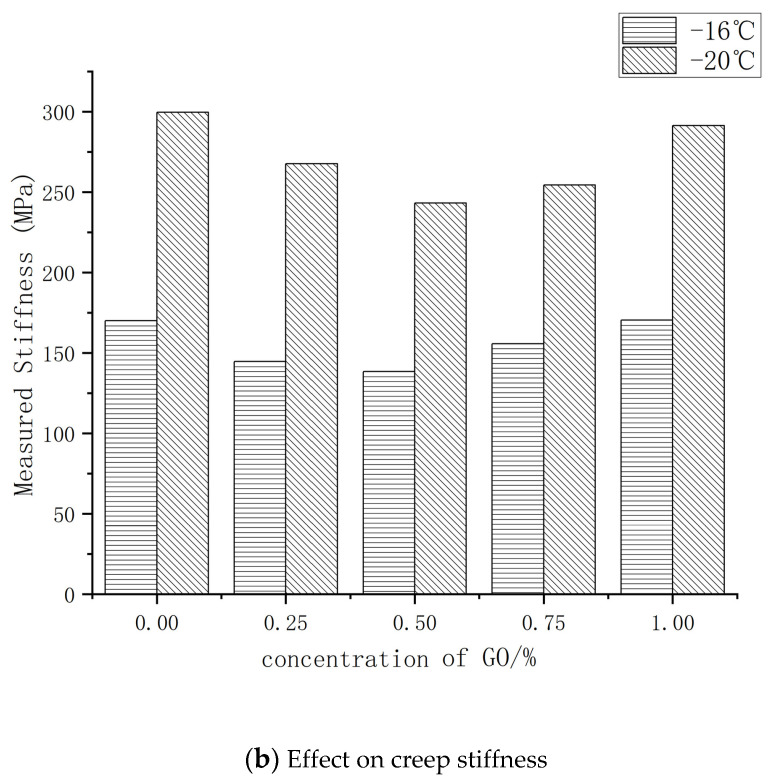
Effect of GO addition on the low-temperature cracking parameters of asphalt.

The low-temperature cracking creep performance of the asphalt was tested using a bending beam rheometer, and the results are shown in Figure 7. The low temperature cracking resistance of the modified asphalt is related to the creep stiffness *S* and creep rate *m* of the asphalt binder. As can be seen in Figure 7b, the creep stiffness S showed a decreasing trend that then increased with increasing GO doping at −16 °C and −20 °C, with the lowest value occurring at 0.5% GO doping. The influence of GO doping on the creep stiffness *S* fluctuates widely at both temperatures. As can be seen in Figure 7a, the creep rate *m* shows an increasing trend that then decreases at −16 °C and −20 °C, with a peak at 0.5% GO doping. However, at −20 °C, the fluctuation range of the effect of GO doping on the creep rate *m* is significantly larger than at −16°C, indicating that the effect of GO doping on the creep rate *m* is more significant at lower temperatures. At −16 °C, the creep stiffness of the GO-doped asphalt was significantly lower than at −20 °C. The creep rate was the opposite of the creep stiffness, indicating that the low-temperature crack resistance of the GO-doped asphalt at −16 °C was significantly higher than at −20 °C.

GO doping can thus improve the low-temperature cracking creep performance of the modified asphalt and enhance its stress relaxation ability, preventing low-temperature cracking behavior. At the same time, the best improvement of the low-temperature cracking resistance of the TPU–SBS-modified asphalt was achieved at a GO doping of 0.5%.

### 3.3. Effect of Graphene Oxide (GO) on the Low-Temperature Performance of TPU–SBS Asphalt Mixtures

**Figure 8:**
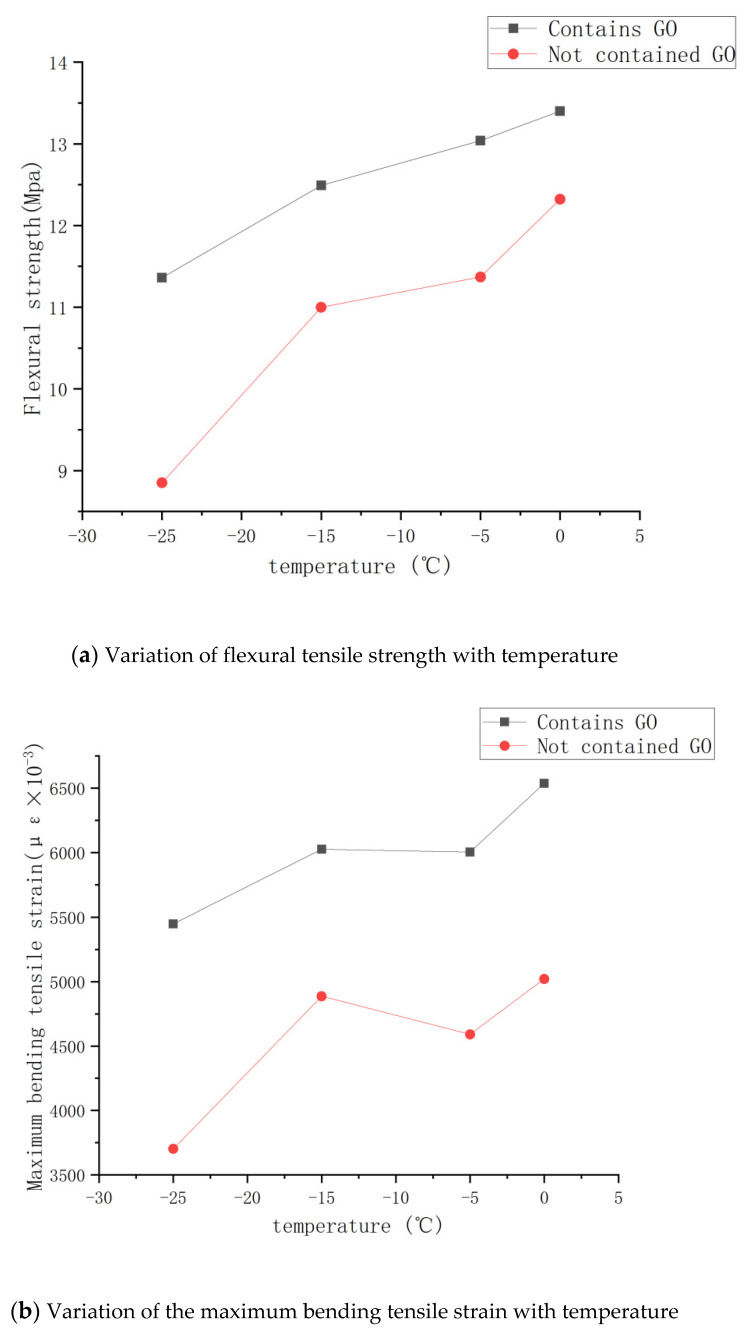

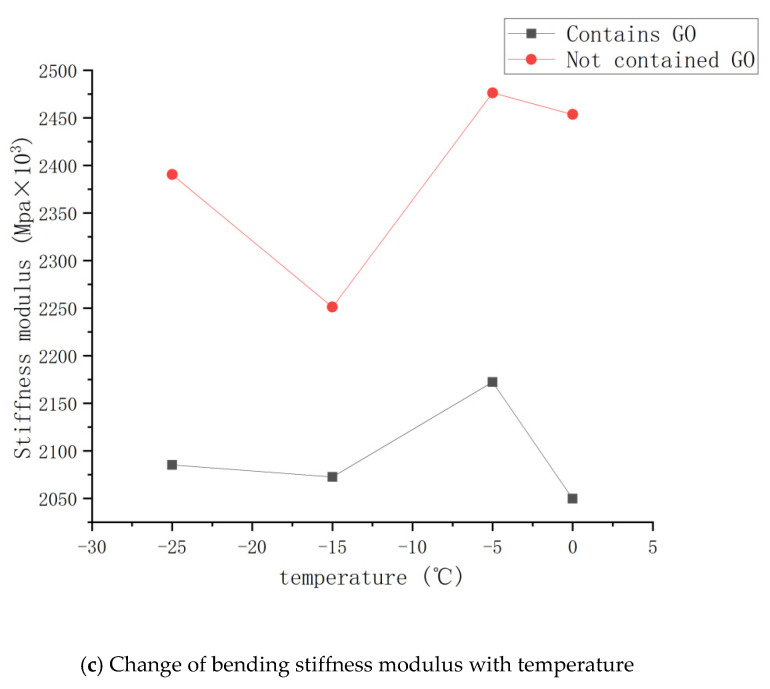
Variation diagram of the performance index with temperature.

According to the results of the bending beam rheological test, the best low-temperature crack resistance of the composite-modified asphalt was obtained at 0.5% GO doping, which was thus selected as the representative value asphalt for the bending test. The effects of GO on the low-temperature crack resistance of the asphalt mixture were analyzed by the bending test, and are shown in Figure 8.

The bending tensile strength of the blend containing GO was 11.36, 12.49, 13.04, and 13.4 MPa at −25 °C, −15 °C, −5 °C, and 0 °C, respectively. Compared with the blend without GO, the bending tensile strength, respectively increased by 28.36%, 13.54%, 14.68%, and 8.76%. The bending and tensile strengths had increased by 28.36%, 13.54%, 14.68%, and 8.76%, respectively, compared with the GO-free mix. Therefore, GO can significantly increase the flexural tensile strength of the mixes and improve their ability to resist temperature shrinkage stresses.

The maximum bending tensile strain is an indicator of the low-temperature deformation of asphalt mixes [27,28,29]_._ The higher the maximum bending tensile strain, the better the low-temperature crack resistance of the mix. The maximum bending tensile strains of the mixes containing GO were 5448.95, 6025.99, 6003.06, and 6536.61 με at −25 °C, −15 °C, −5 °C, and 0 °C, respectively. The maximum bending tensile strains at each low temperature were significantly greater than those of the mixes without GO. This is due to how the GO nanomaterial forms a more stable structure in the mix, improving the crack resistance of the mix.

The bending modulus of rigidity can, to some extent, reflect the ease of the low-temperature cracking of the asphalt mixes. The smaller the bending stiffness modulus, the greater the low-temperature cracking energy required for the mix, and the better the resistance to cracking [28]. As can be seen from Figure 8c, the bending stiffness modulus of the mixes containing GO was significantly lower than that of the mixes without GO at the same temperature.

At the same time, the range and slope of the curve changes in the three graphs show that the incorporation of GO makes the mix less sensitive to low temperatures and improves its resistance to low-temperature cracking.

### 3.4. OpenCV Image Characterization of the Effect of Graphene Oxide (GO) on the Low-Temperature Cracking of the TPU–SBS Asphalt Mixes

For the low-temperature cracking of a small beam, a typical temperature of −15 °C was chosen. The OpenCV image processing process can be divided into on-site and off-site steps [30]. In the on-site step, the cracking of the beam is recorded by a camera mounted on a stand during a three-point loading test. The duration of the recording is from the start of the loading until the beam cracks and damage occurs. The recorded video is then transferred from the temporary memory of the camera to the permanent memory of the computer hard drive. The frame rate of the original video is maintained during the transfer, and no compression of the video is required.

In off-site image processing, the test video is first backgrounded and then a suitable threshold is selected to convert the video into a binary image. The scanning software is then used to check the parameters so that the video meets the applicability requirements. The test process was selected as typical when the beam was first loaded, when the beam was initially cracked by the load, and when the beam was completely damaged. The selected typical processes were processed by OpenCV pixel subtraction using Python, and a comparison exercise was carried out after filtering the contours.

**Figure 9:**
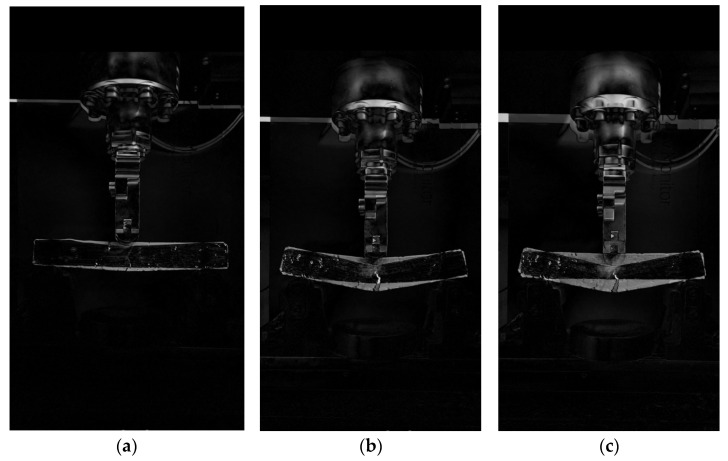
Cracking process diagram of trabecular with GO. (**a**) Comparison between when the beam is first loaded and when the beam is initially cracked by the load; (**b**) Comparison of the initial development of cracks when the beam is subjected to load and when the beam is completely damaged; (**c**) Comparison between when the small beam is first loaded and when the small beam is completely destroyed.

**Figure 10:**
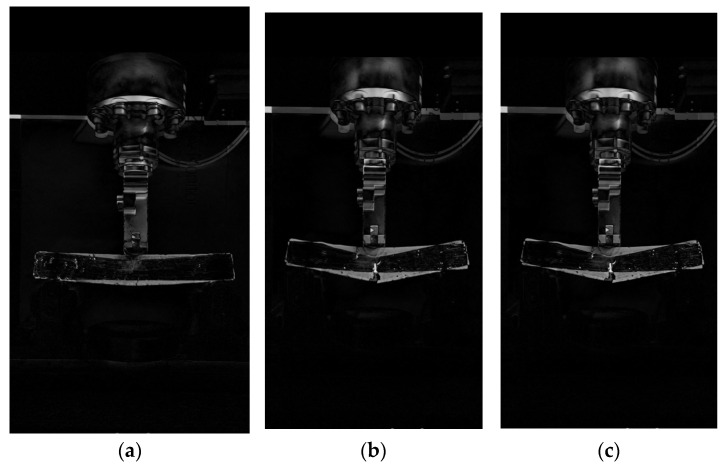
Cracking process diagram of trabecular without GO. (**a**) Comparison between when the beam is first loaded and when the beam is initially cracked by the load; (**b**) Comparison of the initial development of cracks when the beam is subjected to load and when the beam is completely damaged; (**c**) Comparison between when the small beam is first loaded and when the small beam is completely destroyed.

The results are shown in Figure 9 and Figure 10, with (a) showing the comparison between the initial loading of the beam and the initial cracking of the beam under load, (b) showing the comparison between the initial development of the crack when the beam is subjected to load and when the beam is completely damaged, and (c) showing the comparison between the beam at the beginning of loading and at complete failure. From (a), it can be seen that the deflection of the GO-modified beam at the initial crack initiation is less than that of the beam without the GO modifier. From (b) and (c), it can be seen that the depth of crack development and the deflection at the failure of the GO-modifier-doped beam is less than that when loaded to complete failure. It is clear from the treatment results graph that the low-temperature performance of the GO-modified asphalt mix beams is significantly better than that of the unadulterated GO-modified asphalt mix beams. This implies that GO incorporation can improve the low-temperature cracking resistance of the modified asphalt mix and can effectively avoid the low-temperature cracking behavior of the asphalt mix.

## 4. Conclusions

From the test results and analysis, the conclusions of this study are summarized below.

(1) Graphene oxide (GO) is present in the composite as folded flakes, and its presence increases the degree of surface folding of the asphalt matrix, which can make the asphalt matrix less temperature-sensitive and make the asphalt polymer more stable.

(2) Graphene oxide (GO) has the best effect on improving the low-temperature crack resistance of TPU–SBS-modified asphalt at a doping ratio of 0.5%.

(3) The addition of graphene oxide (GO) significantly improved the low-temperature crack resistance of TPU–SBS-modified asphalt and enhanced the stress relaxation ability of the asphalt, making it more suitable for construction in cold areas and areas with large temperature differences between day and night.

(4) The introduction of OpenCV images allowed for the clearer expression of the improvement effect of graphene oxide (GO) on the low-temperature crack resistance of asphalt mixes.

The results of the study showed that the incorporation of GO improved the low-temperature crack resistance of the asphalt compared with TPU–SBS-modified asphalt. It is expected that the results of the current study will encourage further research into the use of GO modifiers for the production of modified asphalt mixtures and promote their application in cold zone road construction.

## Figures and Tables

**Table 1:** Technical specifications for the 90-base asphalt used in this study.

	Test Results	Technical Requirements (Reference GTGF40-2004)
Penetration (0.1 mm)	87.7	80–100
Softening point (Global Method) (°C)	47.2	≥45
Ductility (cm)	46.5	≥45
Solubility (%)	102.3	≥99.9
Dynamic viscosity at 60 °C (Pa∙s)	177.7	≥160

**Table 2:** Technical specifications for the SBS used in this study.

Type	S/B Ratio	Oil Filling Rate (%)	Volatile Components (≤%)	Ash (≤%)	300% Tensile Stress (≥MPa)	Tensile Strength (≥MPa)	Elongation at Tear (≥%)	Tear off Permanent (≤%)	Shore Hardness (A)	Melt Flow Rate (g/10 min)
SBSYH-792E	40/60	0	0.7	0.2	3.5	24	730	55	≥85	0.10–5.00

**Table 3:** Technical indicators of the GO used in this research.

	Symbol	Detection of Typical Values
Place of Origin		Qitaihe City, Heilongjiang Province, China
Place of Origin		Black powder
Oxygen level	%	44.88
Particle size	D50 (μ)	30.22
Thickness	(μh)	<5
Specific surface area	m^2^·g^−1^	>500

**Table 4:** Mineral aggregate gradation of the asphalt mixture.

Particle Size	10–20 mm	5–10 mm	3–5 mm	0–3 mm	Mineral Powder	Upper Grading Limit	Lower Grading Limit	Median Gradation	Composite Gradation
19	100	100	100	100	100	100	100	100	100
16	86.1	100	100	100	100	100	90	95	97.1
13.2	45.8	100	100	100	100	92	76	84	88.6
9.5	0.74	94.2	100	100	100	80	60	70	76.9
4.75	0.5	8.5	92.1	100	100	62	34	48	42.9
2.36	0	0.5	11.1	89.4	100	48	20	34	30.6
1.18	0	0	3.5	68.2	100	36	13	24.5	23.1
0.6	0	0	1.6	51.7	100	26	9	17.5	17.7
0.3	0	0	0	36.1	99	18	7	12.5	12.7
0.15	0	0	0	27.1	94.4	14	5	9.5	9.7
0.075	0	0	0	16.8	75.7	8	4	6	6.2

## Data Availability

The data of this study have been included in the manuscript.
